# Supplementary material for: Luminescent Molecularly Imprinted Polymers Based on Covalent Organic Frameworks and Quantum Dots with Strong Optical Response to Quinoxaline-2-Carboxylicacid
Source: Polymers (Basel). 2019 Apr 17;11(4):708. doi: 10.3390/polym11040708 (PMC6523229; doi:10.3390/polym11040708)
Supplement: Supplementary file 1 [file polymers-11-00708-s001.pdf]

Supplementary materials

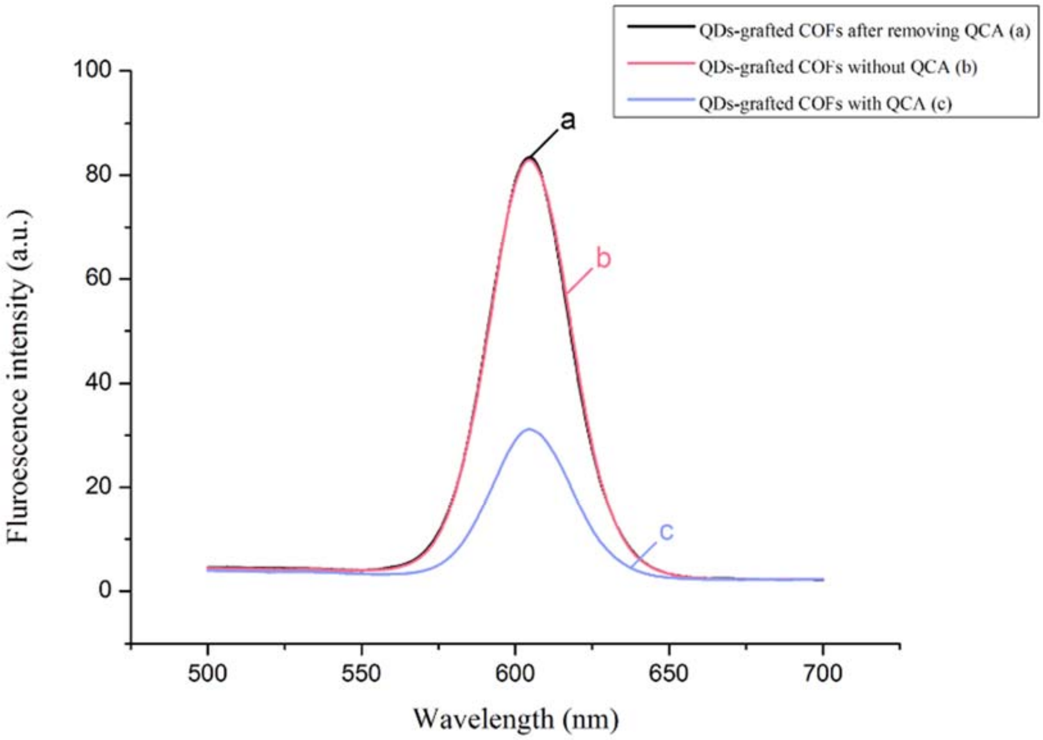

Figure S1. Fluorescence spectra of (a) QDs-grafted COFs after removing QCA, (b) QDs-grafted COFs without QCA, (c) QDs-grafted COFs with QCA.

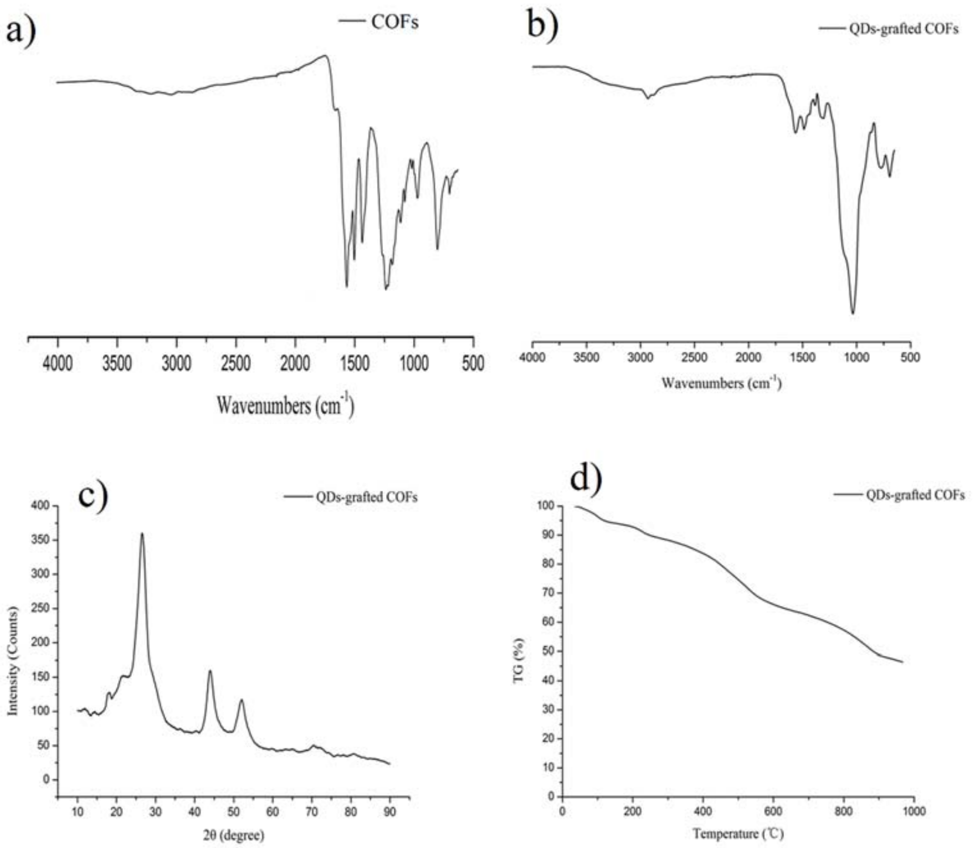

Figure S2. (a) FT-IR spectrum of COFs, (b) FT-IR spectrum of QDs-grafted COFs, (c) XRD spectrum of QDs-grafted COFs, (d) TGA plot of QDs-grafted COFs.

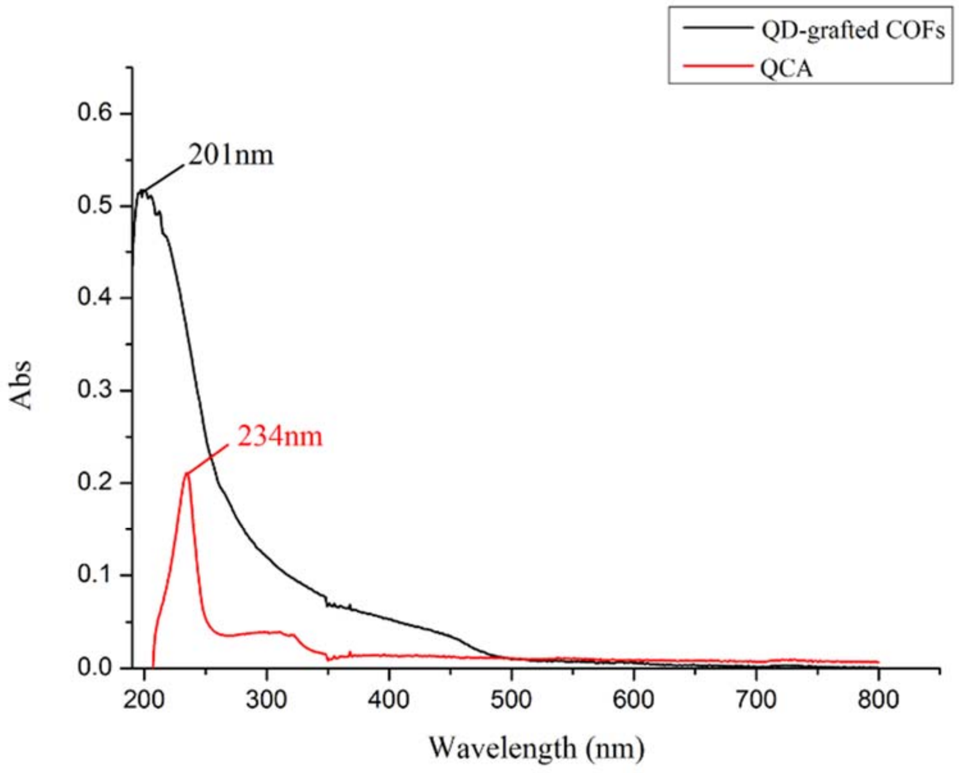

Figure S3. UV-vis spectra of (a) QCA solution and (b) QDs-grafted COFs.

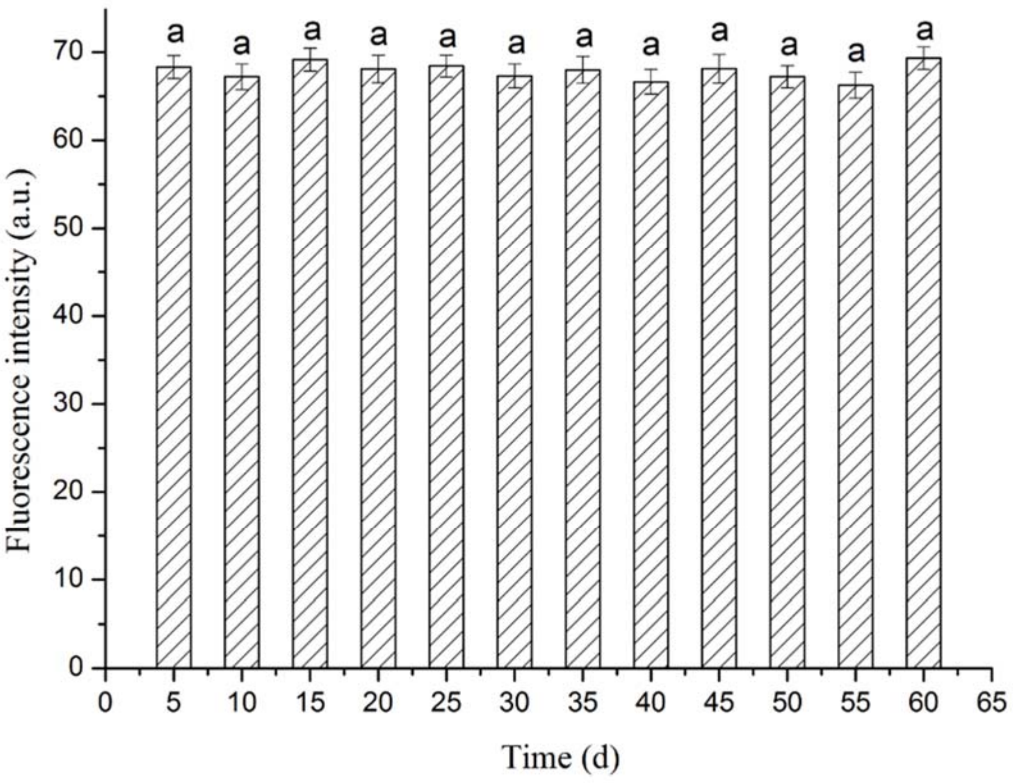

Figure S4. Photostabilities of QDs-grafted COFs.
